# Enhanced Integration of TB Services in Reproductive Maternal Newborn and Child Health (RMNCH) Settings in Eswatini

**DOI:** 10.1371/journal.pgph.0000217

**Published:** 2022-04-20

**Authors:** Kieran Hartsough, Chloe A. Teasdale, Siphesihle Shongwe, Amanda Geller, Eduarda Pimentel De Gusmao, Phumzile Dlamini, Arnold Mafukidze, Munyaradzi Pasipamire, Trong Ao, Caroline Ryan, Surbhi Modi, Elaine J. Abrams, Andrea A. Howard

**Affiliations:** 1 ICAP-Columbia University, Mailman School of Public Health, New York, NY, United States of America; 2 CUNY Graduate School of Public Health & Health Policy, Department of Epidemiology & Biostatistics, New York, NY, United States of America; 3 ICAP-Columbia University, Mailman School of Public Health, Mbabane, Eswatini; 4 US Centers for Disease Control and Prevention (CDC), Division of Global HIV & TB, Atlanta, GA, United States of America; 5 ICAP-Columbia University, Mailman School of Public Health, Maputo, Mozambique; 6 Center for Global Health Practice and Impact, Georgetown University, Mbabane, Eswatini; 7 CDC-Eswatini, Division of Global HIV & TB, Mbabane, Eswatini; 8 Department of Epidemiology, Mailman School of Public Health, Columbia University, New York, NY, United States of America; Amsterdam Institute for Global Health and Development, NETHERLANDS

## Abstract

Tuberculosis (TB) primarily affects women during their reproductive years and contributes to maternal mortality and poor pregnancy outcomes. For pregnant women living with HIV (WLHIV), TB is the leading cause of non-obstetric maternal mortality, and pregnant WLHIV with TB are at increased risk of transmitting both TB and HIV to their infants. TB diagnosis among pregnant women, particularly WLHIV, remains challenging, and TB preventive treatment (TPT) coverage among pregnant WLHIV is limited. This project aimed to strengthen integrated TB and reproductive, maternal, neonatal and child health (RMNCH) services in Eswatini to improve screening and treatment for TB disease, TPT uptake and completion among women receiving RMNCH services. The project was conducted from April-December 2017 at four health facilities in Eswatini and introduced enhanced monitoring tools and on-site technical support in RMNCH services. We present data on TB case finding among women, and TPT coverage and completion among eligible WLHIV. A questionnaire (S1 Appendix) measured healthcare provider perspectives on the project after three months of project implementation, including feasibility of scaling-up integrated TB and RMNCH services. A total of 5,724 women (HIV-negative or WLHIV) were screened for active TB disease while attending RMNCH services; 53 (0.9%) were identified with presumptive TB, of whom 37 (70%) were evaluated for TB disease and 6 (0.1% of those screened) were diagnosed with TB. Among 1,950 WLHIV who screened negative for TB, 848 (43%) initiated TPT and 462 (54%) completed. Forty-three healthcare providers completed the questionnaire, and overall were highly supportive of integrated TB and RMNCH services. Integration of TB/HIV services in RMNCH settings was feasible and ensured high TB screening coverage among women of reproductive age, however, symptom screening identified few TB cases, and further studies should explore various screening algorithms and diagnostics that optimize case finding in this population. Interventions should focus on working with healthcare providers and patients to improve TPT initiation and completion rates.

## Introduction

Globally, among the 10 million cases of tuberculosis (TB) in 2019 there were 3.2 million cases in women and 1.2 million in children [1]. TB primarily affects women during their reproductive years (15-49 years) and contributes significantly to maternal mortality and poor pregnancy outcomes [2, 3]. TB is a leading cause of death from an infectious disease, especially among people living with HIV (PLHIV) [4]. For pregnant women living with HIV (WLHIV), TB is the leading cause of non-obstetric maternal mortality [5], and pregnant WLHIV with TB are at increased risk of transmitting both TB and HIV to their infants [6]. Early diagnosis of TB and provision of TB preventive treatment (TPT) for PLHIV and child contacts of cases are key components of the End TB Strategy [7], with TPT shown to reduce TB incidence when combined with antiretroviral therapy (ART) and independently improve survival among PLHIV [8–11]. However, identification of TB disease among pregnant women remains challenging as TB symptoms may be similar to the physiological symptoms of pregnancy, and TPT coverage among pregnant WLHIV has been limited, primarily due to concerns about toxicity [12, 13].

Eswatini has one of the world’s highest TB/HIV burdens; in 2019, the TB incidence rate was 363 cases per 100,000 people and 66% of TB patients with known HIV status were HIV-positive [1]. The estimated prevalence of HIV in Eswatini among women of reproductive age was 34% in 2017 [14]. National guidelines call for TB screening at all visits to a health facility (intensified case finding), with provision of TPT for all PLHIV without TB disease, including pregnant women and children over one year of age, and all child contacts of people with bacteriologically-confirmed pulmonary TB [15]. TPT coverage has greatly improved, but still has limitations with 32% of child contacts of known TB cases receiving TPT in 2019 [1].

This paper describes a project to strengthen integrated TB and reproductive, maternal, neonatal and child health (RMNCH) services in Eswatini for pregnant and postnatal women, as well as women accessing family planning (FP) services, and their children. We present the service implementation design and project data, including TB case finding, and TPT coverage and completion among eligible HIV-positive women in key entry points to health services. We also describe provider perspectives on the acceptability and feasibility of implementing integrated TB services in RMNCH services.

## Methods

In coordination with the Eswatini Ministry of Health (MOH), ICAP at Columbia University and the US Centers for Disease Control and Prevention (CDC), implemented the project from April through December 2017 at four purposively selected health facilities in the Manzini region of Eswatini. Facilities were chosen if they provided RMNCH and TB services, including on-site access to Xpert MTB/RIF for TB diagnosis. The aim of the project was to strengthen and enhance the integration of routine TB and RMNCH services per Eswatini’s national guidelines, which included TB screening at health facilities conducted at the entrance or within clinics by cough monitors (lay health workers responsible for performing TB screening for all patients and sputum collection for those identified with presumptive TB) [16].

The project included introduction of enhanced monitoring tools and on-site technical support to improve screening and treatment for TB disease, TPT uptake and completion, and TB contact tracing of children under 5 years among women receiving RMNCH services including antenatal care (ANC), postnatal care (PNC) and FP services at the four facilities. At the time of the project, the health facilities in Eswatini were using the existing MOH Presumptive TB Register which only captured data on patients with a positive TB screen. For the project, an enhanced RMNCH TB register was introduced at the ANC, PNC, and FP points of service at each of the four facilities. The enhanced RMNCH TB register was a longitudinal, paper-based register designed to capture indicators along the entire TB cascade including screening (positive or negative), investigation, diagnosis, treatment, or prevention, and to track initial and all follow-up visits for each woman accessing ANC, PNC, and FP services within each of the service areas (women receiving services across multiple service areas were entered in multiple registers). In addition, contact tracing was strengthened through the introduction of a Contact Tracing Tool added to all index case files, and supported integration of a new MOH Contact Tracing Register into RMNCH services, to allow for identification and longitudinal follow-up of all partner and child contacts of TB cases including screening, laboratory testing, treatment and TPT information for contacts. As part of the enhanced technical support, ICAP trained RMNCH staff, including nurses, cough monitors and peer mentor mothers on TB screening and following women and children through the TB cascade to ensure appropriate preventive or curative treatment, and provided ongoing monitoring and supportive supervision during the implementation period. All staff were trained on use of the registers and study procedures. Weekly visits were conducted by an ICAP Nurse Mentor who reviewed register completion and data and provided data-driven clinical mentoring to RMNCH staff. No additional health facility staff were hired as part of the project, nor were additional TB diagnostic supplies provided.

An existing Eswatini MOH TB screening tool that included key signs and symptoms of TB (current cough, fever for two weeks or more, night sweats for two weeks or more, unintended weight loss or poor weight gain in pregnant women and children, and history of contact with a TB case in children only) was used in the project. At project sites, screening results were recorded in the enhanced RMNCH TB Register. All RMNCH patients who screened positive for TB symptoms were asked to provide a sputum sample to a facility cough monitor, with samples taken immediately to the on-site laboratory for testing with Xpert MTB/RIF assay (Cepheid, Sunnyvale, CA, US). Cough monitors returned Xpert results to patients and escorted those diagnosed with TB to the TB clinic for same-day treatment initiation, while WLHIV without evidence of active TB were offered TPT (daily isoniazid for 6 months) if deemed eligible by nurses. Active TB disease was assessed by facility clinicians based on national guidelines, which allow for bacteriological confirmation or clinical diagnosis. TPT initiation and follow-up in RMNCH care was newly implemented at the selected health facilities through the project. TPT completion was documented once all refills were administered as per Eswatini national guidelines. For child contacts of adults with TB disease, caregivers were contacted and asked to bring the child to the RMNCH clinic where the MOH TB screening tool was used to identify those requiring diagnostic evaluation. Any contacts diagnosed with TB were to be escorted to the onsite TB clinic, and those without active TB disease were offered TPT (daily isoniazid for 6 months) within RMNCH services, according to national guidelines. Additionally, all child contacts of WLHIV with unknown HIV status were offered HIV testing within RMNCH services following existing national HIV testing and counselling guidelines.

For the evaluation, patient-level data were extracted from the enhanced RMNCH TB registers and the Contact Tracing registers by trained data collectors following the end of the implementation period. Data collectors retrospectively abstracted information for all women documented in the RMNCH TB registers at each point of care (ANC, PNC, FP) during the implementation period (April – December 2017) and transcribed it into an electronic study database. Clients were not de-duplicated across points of care within a health facility. Data collected included date of registration in the RMNCH TB register, age, gestational age for ANC clients, HIV status at entry, HIV testing information for those not known to be positive, ART status, cotrimoxazole (CTX) treatment status and start date, dates and results of all TB screening(s) (positive symptom screen or negative; data on which symptoms a client reported were not available), dates and type of TB diagnostic testing and TB test results, TB diagnosis date, date of TB treatment initiation and treatment outcome, and date of TPT initiation, dates of TPT refills and TPT outcome. Time was provided prior to data collection to allow for TB treatment and TPT completion. For women who were diagnosed with TB, information on child contacts under 5 years was collected from the Contact Tracing Registers, including the date the index case started treatment, index case TB type (drug-resistant or not), sex and age of the child, child’s TB screening result, date sputum collected or referral for sputum collection, results of TB test, whether the contact was eligible for TPT, date of TPT initiation, results of repeat TB screening at 3, 6 and 9 months, and TPT outcome.

To assess proportions of women receiving screening and other TB-related services, data on the number of women who accessed ANC and PNC services during the implementation period were obtained from the health facility ANC and PNC registers, respectively, while data on the number of women who accessed FP services during the period were not available. We present descriptive statistics for women in ANC, PNC and FP services, and an analysis of the TB/HIV cascade, including the proportion of women attending ANC and PNC services who were screened for TB. Cochran-Mantel-Haenszel tests and generalized linear models, adjusted for facility clustering, were used to compare differences in proportions and medians, respectively, between women initiated on TPT and those not initiated on TPT. We also summarized data on TB diagnosis, TPT initiation and completion, and contact tracing results.

In addition, an anonymous and voluntary self-administered tablet-based questionnaire was used to measure healthcare provider perspectives on the project after three months of project implementation, including feasibility of scaling-up integrated TB and RMNCH services. The self-administered questionnaire was offered in English and Siswati (following verbal consent) to all healthcare providers, including cough monitors, nurses, and clinicians, working within RMNCH services at the time of survey administration. Verbal consent was used rather than written consent as the questionnaire involved no more than minimal risk to subjects and ensured that no identifying information was collected, and respondents remained anonymous. Informed consent forms were available in English and SiSwati and participants were offered a copy of the consent form for their records. Data collectors addressed questions raised by respondents before administering the questionnaire. Once the content of the informed verbal consent had been reviewed completely, healthcare providers were given an electronic tablet to complete the questionnaire, and prior to the questionnaire launching were asked on the electronic tablet if they understood the project and whether they agreed to participate. We report descriptive statistics including frequency of responses for each quantitative item as well as identification of themes for qualitative items. All analyses were conducted with SAS 9.4.

### Ethics statement

The project evaluation was approved by the Columbia University Irving Medical Center Institutional Review Board, and the Eswatini Health and Human Research Review Board, which both waived the requirement for informed consent and approved the use of verbal consent for the healthcare provider interviews. The protocol was also reviewed in accordance with CDC human subjects protections procedures and was determined to be research, but CDC investigators were not engaged and did not interact with human subjects or have access to identifiable data or specimens for research purposes, and the funders had no role in data collection or analysis.

## Results

### TB screening

Between April and December 2017, a total of 3,361 (80%) out of 4,199 women receiving ANC or PNC services were screened for TB at least once at the four participating health facilities. By service point, TB screening coverage was 99% (2,096/2,125) in ANC and 61% (1,265/2,074) in PNC. In addition, 2,363 women receiving services in FP were screened for TB (denominator unknown), for a total of 5,724 women screened over the implementation period.

Of those screened for TB, 1,977 (35%) were HIV-positive (known or newly diagnosed), of whom 94% were on ART (currently or newly initiated). Characteristics of women screened for TB while attending RMNCH services are shown in Table 1.

**Table 1:** Characteristics of women screened for TB while attending RMNCH services by point of care at four health facilities in Eswatini, 2017 (N = 5,724).

	Total	Antenatal Care	Postnatal Care	Family Planning
**N**	**5,724**	**2,096**	**1,265**	**2,363**
Median age (IQR), years	27 (22-32)	26 (21-31)	26 (22-31)	28 (23-33)
Median gestational age (IQR), weeks		23 (16-29)	--	--
HIV status, N (%)				
Known positive	1,945 (34)	654 (31)	612 (48)	679 (29)
Currently on ART, N (%)	1,836 (94)	629 (96)	589 (96)	618 (91)
New positive	32 (1)	20 (1)	0 (0)	12 (1)
Newly Started on ART, N (%)	17 (53)	14 (70)	0	3 (25)
Positive Total	1,977 (35)	674 (32)	612 (48)	691 (30)
Negative	3,584 (63)	1,346 (64)	629 (50)	1,609 (68)
Unknown	163 (3)	76 (4)	24 (2)	63 (3)
Cotrimoxazole, N (%)*	1,537 (78)	584 (87)	480 (78)	473 (68)

*Percentage among known and new HIV positive women

### Intensive TB case finding cascade

Of 5,724 women who were screened for TB while attending RMNCH services, 53 (0.9%) were identified with presumptive TB, including 32 (1.5%) in ANC, 8 (0.6%) in PNC and 13 (0.6%) in FP services. Among those with presumptive TB, 27 (51%) were HIV-positive.

Of the 53 women with presumptive TB, 37 (70%) were evaluated for TB disease, 35 (94%) with Xpert MTB/RIF, 1 (3%) with culture and 1 (3%) with chest radiograph. Of those evaluated for TB, 6 (16%) women (0.1% of those screened), all identified in ANC, were diagnosed with TB, including 3/1,945 HIV-positive women (154 per 100,000) and 3/3,779 HIV-negative/unknown status women (79 per 100,000). All 6 women were initiated on TB treatment, and 3 were documented as cured, while the outcomes of the remaining 3 women were undocumented.

### TPT cascade for HIV-positive women

**Fig 1:**
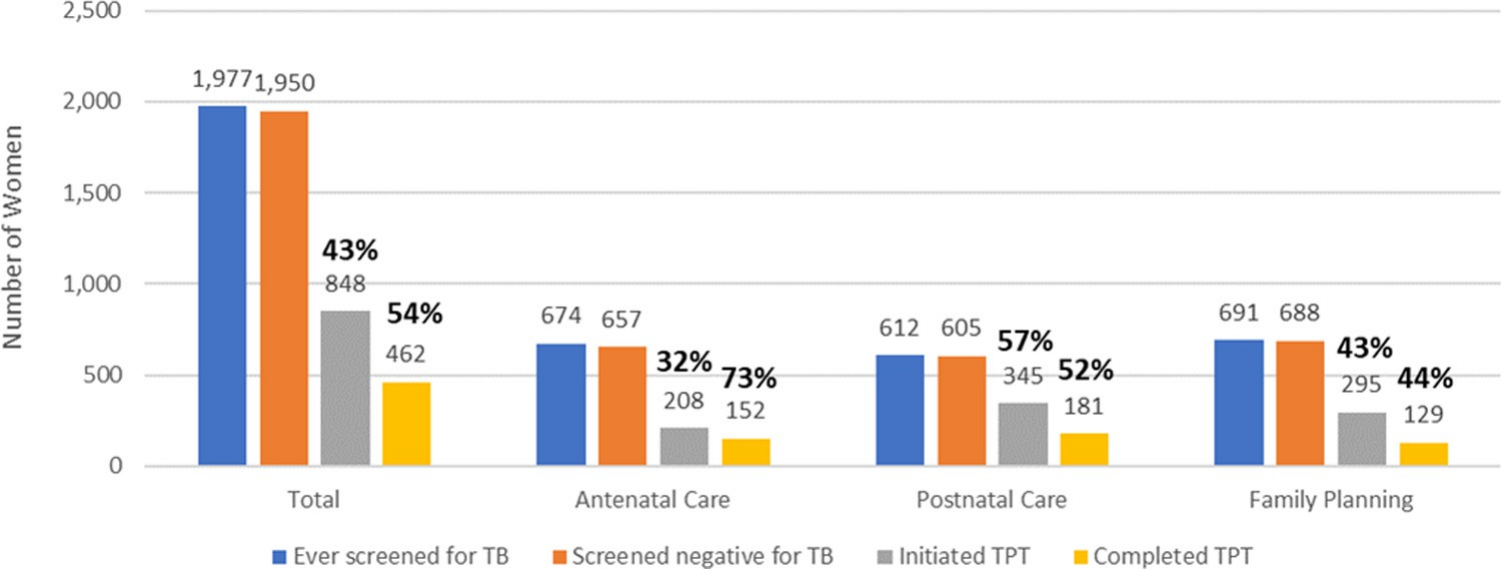
TB Preventive Treatment (TPT) cascade among HIV-positive women at four health facilities in Eswatini, 2017 (N = 1,977)*. *Percentages shown are percent initiated TPT among those that screened negative for TB, and percent completed a course of TPT among those that started a course of treatment.

Among 1,950 HIV-positive women attending RMNCH services who screened negative for TB, 848 (43%) initiated TPT (range across facilities 15-52%), and 462 (54%) completed TPT (range across facilities 43-64%) (Fig 1). A higher proportion of HIV-positive women started TPT in PNC (57%), compared to ANC (32%) and FP (43%), although differences by point of care were not statistically significant (p = 0.5336). TPT completion was highest among women in ANC (73%), while completion was 52% among women in PNC, and 44% among those in FP services. Overall, 24% of women eligible for TPT – those who screened negative for TB – completed a course of TPT (23% in ANC, 30% in PNC, and 19% in FP services).

HIV-positive women who initiated TPT were more likely to be on ART (97% versus 90%, p<.0001) and on cotrimoxazole ((87% versus 71%, p<.0001) compared to women who did not initiate TPT (Table 2).

**Table 2:** Characteristics of HIV-positive women with a negative TB screen by TB Preventive Treatment (TPT) initiation status at four health facilities in Eswatini, 2017 (N = 1,950).

	Total		Initiated TPT		Did not initiate TPT		*p* *
	N	%	N	%	N	%	
	1,950		848	43%	1,102	57%	
**Median (IQR) Age, years**	29	(25-33)	29	(25-33)	29	(25-34)	0.5562
**Median (IQR) Gestational Age (ANC only), weeks**	23.5	(16-29)	24	(17-32)	22	(16-28)	
							0.0004
**On ART (current)**	1,813	93%	824	97%	989	90%	<.0001
**Cotrimoxazole use**	1,517	78%	740	87%	777	71%	<.0001

* Global p-value representing the test for group-level differences comparing those who initiated ART and those who did not by characteristic.

### TB contact tracing

Among the 6 women diagnosed with TB, a total of 10 child contacts less than 5 years old were identified (2 were less than 1 year, and 8 were 1-4 years of age). HIV status information was missing for all contacts, and 8 of the 10 (80%) contacts were brought to the facility and screened negative for TB, while two child contacts 1-4 years of age were not brought for TB screening. Of the 10 child contacts, 3 were contacts of index patients with multi-drug resistant TB, and thus not eligible for TPT per country guidelines. Of the 5 child contacts who were eligible for TPT, all started and completed a course of 6 months of isoniazid.

### Healthcare provider interviews

**Fig 2:**
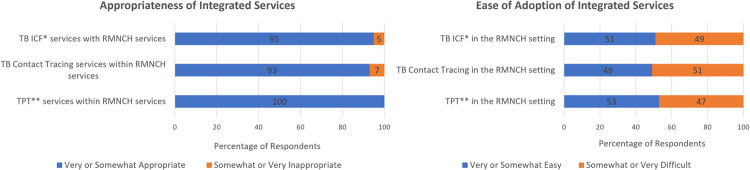
Health care worker perspective on the appropriateness and ease of TB integrated services in reproductive, maternal, neonatal and child health services at four health facilities in Eswatini, 2017 (N = 43). *ICF = Intensified Case Finding, **TPT = Tuberculosis Preventive Treatment.

Among an estimated 45 healthcare providers staffing RMNCH services across the four participating facilities at the time of project implementation, 43 (96%) completed the questionnaire (range across facilities 6-19). HCWs were highly supportive of integration of TB services within RMNCH services, with 100% reporting TB intensified case finding (ICF) was appropriate, 96% reporting TPT integration was appropriate and 93% reporting that TB contact tracing was appropriate (Fig 2). Approximately half of providers reported that this integration was easy to do (53% for ICF, 51% for TPT, 49% for contact tracing). Forty-seven percent of healthcare providers felt that it was very or somewhat easy to adopt the TB RMNCH register and 37% felt that continuing delivery of integrated TB services in RMNCH services was very or somewhat sustainable considering current human resources. Overall, 47% of healthcare providers felt that integrated TB services were a little disruptive and 7% very disruptive to the delivery of routine RMNCH services.

When asked about what they thought were the positive effects of integrating TB services within RMNCH services, healthcare providers reported a common theme of prevention and decrease of new TB infections (49% of respondents). One healthcare provider responded that “*Screening clients for TB makes it easy to identify risk cases (and) therefore it helps in preventing TB from spreading*.*”* In terms of the negative effects of integrating TB services within RMNCH services, healthcare providers most frequently reported the increased workload (47%); as one healthcare provider stated, *“There is usually one nurse in a room which makes it impractical to do the work*. *Either you end up not documenting well or patients end up complaining because of the long queues*.*”* Healthcare providers also reported slower service delivery (21%), with one healthcare provider stating, *“At lot of time is lost in updating the registers thus patients are delayed*.*”* In addition, some healthcare providers had concerns about isoniazid side effects (14%), with one healthcare provider stating, *“I think initiating pregnant (women) on IPT [isoniazid preventive therapy] is a bit difficult because pregnancy on its own has many effects happening to the women then IPT also comes with some side effects*.*”*

When healthcare providers were asked how the integration of TB services within RMNCH services could be improved, a common theme was limiting paperwork and incorporation of the TB/RMNCH register into the existing electronic client management information system (CMIS), as “*Having a different register leads to duplication of some client’s information thus increasing the workload to nurses*, *I would recommend integration of the data tools*.*”* Healthcare providers also felt that hiring of additional staff was needed (26%), and that education was essential (12%), with one provider reporting that, *“I think all clinic personnel should be sensitized so that the burden doesn’t only lie on the few but on everyone thus the service can smoothly run*.*”* In addition, patient health education was reported as needed (12%) with one respondent stating that *“Patients have to be taught well on IPT so that they understand (the) importance of taking it*.*”*

## Discussion

This project demonstrates the feasibility, acceptance, and challenges of integrating TB services in RMNCH settings in Eswatini. During nine months of implementation of integrated services, TB screening coverage was high, diagnostic evaluation and identification of TB cases was low, and there was moderate uptake of TPT with poor completion rates. Furthermore, healthcare providers were supportive of integrated services, but felt that additional resources were needed to effectively implement these services.

While Eswatini has a high burden of TB and HIV, recent years have seen a sharp decrease in TB incidence, dropping from a high of 1,190 cases per 100,000 in 2010 to 363 per 100,000 in 2019, and in 2017, during the period of program implementation, incidence was 502 per 100,0000 [4]. This decrease has been attributed to improvements in ICF with task shifting for TB screening, introduction of Xpert MTB/RIF for TB diagnosis, and implementation of TPT and infection prevention and control measures [17, 18], as well as expanded ART coverage, which increased from 34% in 2010 to over 95% in 2019 [19]. Eswatini had an estimated TB case detection rate of 69% in 2019 [1], thus opportunities remain for early identification of individuals with TB through systematic TB screening and diagnosis at expanded entry points.

This project expanded TB services within ANC, PNC, and FP services, prioritizing routine TB screening and early diagnosis of TB. While screening coverage was high, particularly in ANC, coverage was much lower at PNC and further efforts should be aimed at mentoring providers to ensure a high level of TB screening coverage postpartum. Just under 1% of women screened were identified with presumptive TB, and only six women were diagnosed with TB disease as a result of routine screening in RMNCH services. Other studies in similar settings that were conducted prior to the recommendation of ART for all pregnant WLHIV have found a higher proportion of women with presumptive TB. An implementation research study of integrating ICF with ANC and PMTCT services in six clinics in Soweto, South Africa found that any symptom of TB was reported by 23% of HIV-positive and 14% of HIV-negative women who were newly enrolled in ANC [20], while a study from South Africa found that 16% of HIV-positive pregnant women attending ANC reported any TB symptom [21]. The comparatively low identification of women with presumptive TB in our study may indicate that the symptom screening tool was not conducted with fidelity, or that the tool itself has limited sensitivity among this patient population [22].

Several studies have noted deficiencies in symptom-based ICF among HIV-positive pregnant women. A study among HIV-positive women in ANC in Kenya that compared the diagnostic performance of World Health Organization (WHO) symptom screening and rapid diagnostic tests to sputum culture found that symptom screening alone failed to identify more than half of the cases of culture-confirmed pulmonary TB [23]. A study from South Africa that sought to determine the prevalence of pulmonary TB and evaluate the validity of the WHO symptom screen tool among HIV-positive pregnant women found that 73% of HIV-positive pregnant women with culture positive TB did not report any of the symptoms included in the WHO symptom screening tool [21]. A study done in Eswatini in 2015 that assessed the performance of TB screening and diagnostic tests among pregnant and postpartum women found that the national TB symptom screening tool failed to identify women who tested TB-culture positive and had a sensitivity of 0% [22]. TB symptoms, such as weight loss, may be masked during pregnancy, but TB symptom screening has performed poorly in other populations, including among PLHIV on ART [24, 25], and in general has had mixed results in terms of sensitivity [26–28]. Others have suggested enhancing the TB screening process, through addition of questions to the screening tool, such as if a household member has a positive WHO TB symptom screen [23], or inclusion of diagnostics like Xpert MTB/RIF or chest radiograph as part of screening, as has been recommended by the WHO [21, 29]. However, further research is needed to assess the performance of these screening methods in HIV-positive and HIV-negative women of reproductive age.

Our study found low diagnostic coverage with just 70% of women with presumptive TB documented to have been evaluated for TB. In this study, all women with presumptive TB should have had sputum collected within RMNCH services, but some women may not have been able to produce sputum, and there may have been reluctance to use chest radiograph for women who were pregnant. However, among women who were evaluated for TB, use of Xpert was nearly universal, which is consistent with the Eswatini TB/HIV guidelines [30] and due to the fact that all sites included in this evaluation had onsite access to Xpert MTB/RIF testing.

Through this project, national guidelines for provision of TPT were introduced and implemented in RMNCH settings in the selected health facilities. While less than half of eligible HIV-positive women in RMNCH services initiated TPT, comparatively, TPT coverage among PLHIV newly enrolling in care in Eswatini in 2017 was just 1% [31]. Initiation was relatively higher in women in PNC compared to ANC and FP, and lowest in ANC, perhaps indicative of provider and patient reluctance to start TPT during pregnancy. Despite the WHO and Eswatini guidelines both recommending provision of TPT for eligible pregnant women living with HIV, concerns remain among health care providers and patients of the potential harm of preventive medication – most commonly isoniazid – to the mother or fetus [32]. The IMPAACT P1078 study found that the safety of IPT was similar for women if begun during pregnancy or 12 weeks after delivery; however, there was a statistically significant higher risk of a composite measure of adverse outcomes among women who started IPT during pregnancy compared to those who started IPT 12 weeks after delivery [33]. While this was one study, and other observational studies have not found an increased risk of poor maternal or infant outcomes due to IPT use during pregnancy [34–36], doubts remain about its safety as evidenced by limited TPT initiation in ANC in our study and provider feedback.

Healthcare providers were generally positive about integrating TB services within RMNCH services but felt that additional human resources and streamlined documentation were needed to support the services and decrease burden on providers. At high volume sites, task sharing with lay personnel is needed to ensure proper implementation and continuity of interventions. Rather than creating additional tools, like the RMNCH TB register used in this project due to inability to modify the national CMIS, adding select indicators to existing electronic data systems or registers in use can reduce the documentation burden and likely improve data quality and service integration. Additional staffing to support quality documentation of services, such as hiring of data clerks, may be needed to support service delivery and data quality and completeness.

As this project used data initially collected as part of routine clinical care, there were increased levels of missing and incorrect information compared to data collected as part of a research study. As part of ICAP’s support for service implementation at these health facilities, efforts were made to ensure the highest data quality possible, but the analysis still faced limitations related to poor documentation, including a lack of information on women with presumptive TB who did not have documentation of evaluation for TB disease, and complete TB treatment outcomes. Another limitation is that we did not assess to what extent RMNCH services were impacted by the introduction of TB services. A strength is that the project included a large number of women over a 9-month implementation period across three service points.

In conclusion, we found that integration of TB/HIV services in RMNCH settings is feasible and can ensure high TB screening coverage among women of reproductive age, and continuation of TB services for PLHIV. RMNCH services may be leveraged to increase access to TB services for women of reproductive age. Use and modification of existing monitoring tools, and transition to electronic records, should be emphasized to minimize additional burden on health care providers. As symptom screening identified few TB cases, further studies should explore various screening algorithms and diagnostics that optimize case finding in this population. Interventions, including training, mentorship and continuous quality improvement, should focus on working with health care providers and patients to improve TPT initiation and completion rates.

## Supporting information

S1 Appendix Health care provider questionnaire. (PDF)
