# Supplementary material for: Enhanced Integration of TB Services in Reproductive Maternal Newborn and Child Health (RMNCH) Settings in Eswatini
Source: PLOS Glob Public Health. 2022 Apr 20;2(4):e0000217. doi: 10.1371/journal.pgph.0000217 (PMC10021747; doi:10.1371/journal.pgph.0000217)
Supplement: S1 Appendix — (PDF) [file pgph.0000217.s001.pdf]

Appendix A

Health Care Provider Questionnaire

INTRODUCTION: *First, I would like to thank you for agreeing to complete this questionnaire about the integration of TB services with RMNCH/PMTCT services. This questionnaire should take you about 10 minutes to complete. The answers that you provide will help assess the performance of integration of services and help to make recommendations for improving services for women and children. Please answer all questions based on your own experiences and thoughts.*

1. How appropriate is it to integrate TB Intensive Case Finding services with RMNCH services?

Very appropriate, Somewhat appropriate, Somewhat inappropriate, Very inappropriate

2. How practical is it to implement TB Intensive Case Finding services within RMNCH services?

Very practical, Somewhat practical, Somewhat impractical, Very impractical

3. How easy was it to adopt the activities for TB Intensive Case Finding in the RMNCH setting?

Very easy, Somewhat easy, Somewhat difficult, Very difficult

4. How much do you agree or disagree that continuing the delivery of TB Intensive Case Finding services through RMNCH services in the future is useful?

Strongly Agree, Agree, Disagree, Strongly Disagree

5. How appropriate is it to integrate TB Contact Tracing services within RMNCH services?

Very appropriate, Somewhat appropriate, Somewhat inappropriate, Very inappropriate

6. How practical is it to implement TB Contact Tracing services within RMNCH services?

Very practical, Somewhat practical, Somewhat impractical, Very impractical

7. How easy was it to adopt the activities for TB Contact Tracing in the RMNCH setting?

Very easy, Somewhat easy, Somewhat difficult, Very difficult

8. How much do you agree or disagree that continuing the delivery of TB Contact Tracing services through RMNCH services in the future is useful?

Strongly Agree, Agree, Disagree, Strongly Disagree

9. How appropriate is it to integrate IPT services within RMNCH services?

Very appropriate, Somewhat appropriate, Somewhat inappropriate, Very inappropriate

10. How practical is it to implement IPT services within RMNCH services?

Very practical, Somewhat practical, Somewhat impractical, Very impractical

11. How easy was it to adopt the activities for IPT in the RMNCH setting?

Very easy, Somewhat easy, Somewhat difficult, Very difficult

12. How much do you agree or disagree that continuing the delivery of IPT services through RMNCH services in the future is useful?

Strongly Agree, Agree, Disagree, Strongly Disagree

13. How appropriate is it to integrate TB Infection Prevention and Control measures within RMNCH services?

Very appropriate, Somewhat appropriate, Somewhat inappropriate, Very inappropriate

14. How practical is it to implement TB Infection Prevention and Control measures within RMNCH services?

Very practical, Somewhat practical, Somewhat impractical, Very impractical

15. How easy was it to adopt the TB Infection Prevention and Control measures in the RMNCH setting?

Very easy, Somewhat easy, Somewhat difficult, Very difficult

16. How much do you agree or disagree that continuing the delivery of TB Infection Prevention and Control measures through RMNCH services in the future is useful?

Strongly Agree, Agree, Disagree, Strongly Disagree

17. How much do you agree or disagree that conducting TB Services Capacity Assessments are useful to assess integration of TB services with RMNCH services?

Strongly Agree, Agree, Disagree, Strongly Disagree

18. How easy was it to adopt the TB RMNCH register?

Very easy, Somewhat easy, Somewhat difficult, Very difficult

19. Considering current human resources, how sustainable is it to continue delivery of integrated TB services in RMNCH services?

Very sustainable, Somewhat sustainable, Somewhat unsustainable, Very unsustainable

20. How disruptive are integrated TB services to the delivery of routine RMNCH services?

Not at all disruptive, Somewhat disruptive, A little disruptive, Very disruptive

21. What do you think are the positive effects of integrating TB services within RMNCH services?

Text Response

22. What do you think are the negative effects of integrating TB services within RMNCH services?

Text Response

23. How could the integration of TB services within RMNCH services be improved?

Text Response
